# Reinvestigation of Na_5_GdSi_4_O_12_: A Potentially Better Solid Electrolyte than Sodium β Alumina for Solid-State Sodium Batteries

**DOI:** 10.1021/acsami.3c16153

**Published:** 2024-01-31

**Authors:** Anna Michalak, Santosh Behara, Anji Reddy M

**Affiliations:** IMPACT Energy Storage Laboratory, Faculty of Science and Engineering, Swansea University, Fabian Way, Swansea SA1 8EN, U.K.

**Keywords:** solid-state sodium batteries, solid electrolytes, Na_5_GdSi_4_O_12_ (NGS), Na-β″-alumina (BASE), high ionic conductivity, ceramic electrolyte

## Abstract

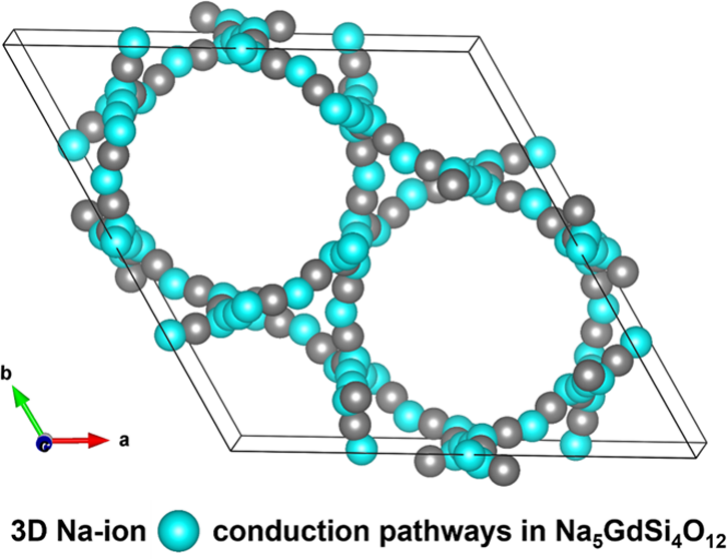

Developing high-performing solid electrolytes that could replace flammable organic liquid electrolytes is vital in designing safer solid-state batteries. Among the sodium-ion (Na^+^) conducting solid electrolytes, Na-β″-alumina (BASE) is highly regarded for its employment in solid-state battery applications due to its high ionic conductivity and electrochemical stability. BASE has long been employed in commercial Na–NiCl_2_ and Na–S batteries. However, the synthesis of highly conductive BASE is energy-intensive, involving elevated temperatures for sintering and the incorporation of stabilizing additives. Additionally, BASE is highly sensitive to humidity, which limits its applications. Hence, there is an intense search to identify suitable high-performing solid electrolytes that could replace BASE. In this context, we reinvestigated Na_5_GdSi_4_O_12_ (NGS) and demonstrated that phase pure NGS could be synthesized by a simple solid-state reaction. Beyond a high ionic conductivity of 1.9 × 10^–3^ S cm^–1^ at 30 °C (1.5 × 10^–3^ S cm^–1^ for BASE), NGS exhibited high chemical as well as electrochemical stability, lower interfacial resistance, lower deposition and stripping potential, and higher short-circuiting current, designating NGS as a better solid electrolyte than BASE.

## Introduction

Sodium-based batteries are one of the most captivating and feasible technologies among the post-lithium electrochemical energy storage devices. Sodium mineral deposits are both highly abundant and widely distributed around the globe, making them a more sustainable and economically promising candidate.^1,2^ However, the high chemical reactivity of sodium with organic liquid electrolytes raises safety concerns.^3^ Grounded on these facts, solid-state sodium batteries (SSSBs) have emerged as a promising alternate system, where flammable liquid electrolytes are replaced with solid electrolytes, offering higher thermal stability and overall system stability against Na-metal.^4,5^ Solid electrolytes play an important role in deciding the overall performance of SSSBs. High ionic conductivity and chemical and electrochemical stability are essential criteria that solid electrolytes should be endowed with for solid-state battery applications.^6^ Among various classes of solid electrolytes (SEs), ceramic-based SEs exhibit most of these properties and, thus, are widely investigated for SSSBs. When sodium-ion (Na^+^) conducting SEs are considered, Na-β″-alumina (BASE) and NASICON-based Na_3_Zr_2_PSi_2_O_12_ (NZPS and doped) are highly regarded for solid electrolyte applications.^6,7^ BASE exhibits ionic conductivity of 10^–2^–10^–3^ S cm^–1^ at room temperature (RT). It is highly stable against Na-metal and has long been used in commercial Na–NiCl_2_ and Na–S cells that operate between 300 and 350 °C.^8^ However, the synthesis of BASE involves several processing steps. Temperatures higher than 1500 °C are required to sinter, and additives are needed to stabilize higher conducting polymorph of BASE.^6,8^ It is also highly sensitive to water or water vapors.^9,10^ On the other hand, the ionic conductivity of NZPS-based compounds ranges from 10^–3^ to 10^–4^ S cm^–1^ at RT.^6^ Similar to BASE, they exhibit high stability against Na-metal^11^ and impressive stability against moisture.^12^ However, synthesizing NZPS compounds on a large scale is a matter of concern.^11^ They also suffer from high interfacial resistance.^6^ Consequently, research and development efforts to identify, design, and formulate suitable Na^+^ conducting solid electrolytes gain prominence.

In pursuit of identifying suitable Na^+^ conducting SEs, Na_5_MSi_4_O_12_-type compounds caught our attention. Maksimov et al. introduced these compounds (Na_5_YSi_4_O_12_, Na_5_ScSi_4_O_12_, and Na_5_ErSi_4_O_12_) in the 1970s.^13^ Shannon et al. were the first to report the ionic conductivity and determine the true crystal structure of these compounds.^14^ These phases generated a lot of interest recently.^15−17^ Shannon et al. prepared a series of compounds by substituting M with various elements (M = Fe, Sc, Y, and rare earth Lu–Sm). They found that the ionic conductivity increased linearly with the ionic radii of the M^3+^ ion in the structure. For example, the ionic conductivity of Na_5_FeS_14_O_12_ is 2 × 10^–5^ S cm^–1^, and Na_5_SmSi_4_O_12_ is 1 × 10^–1^ S cm^–1^ at 200 °C. Sm^3+^ with an ionic radius of 0.958 Å seems to be the higher limit in the series as the compounds with larger M^3+^ ions (Nd^3+^, Pr^3+^, La^3+^) did not form.^14^ Since their discovery, many compounds of these types have been prepared, usually by following a conventional solid-state synthesis.^14,18^ However, other preparation techniques have also been proposed, including glass melt-quenching,^19^ spray freeze-drying,^20^ hydrothermal processes,^14,21^ and sol–gel methods.^22^ Although the Sm compound showed high conductivity, it is unstable toward Na-metal.^14^ Gd compound with an ionic conductivity of 8 × 10^–2^ S cm^–1^ at 200 °C is the second highest conducting compound in this series and stable against Na-metal. Therefore, we chose Na_5_GdSi_4_O_12_ (NGS). While a few reports describe the preparation of single-phase NGS,^20,22−24^ no consistent synthesis process of NGS has been proposed. Further, there are no reports exploring the use of NGS as a solid electrolyte for SSSBs. In this study, we unequivocally demonstrate the synthesis of pure NGS through a simple two-step solid-state reaction, exploring its properties and candidature as a solid electrolyte for SSSBs. We also compared its properties with BASE, “the electrolyte of choice”^25^ for solid-state cells. NGS outperformed BASE as a solid electrolyte in several aspects.

## Synthesis and Ionic Conductivity of NGS

**Figure 1:**
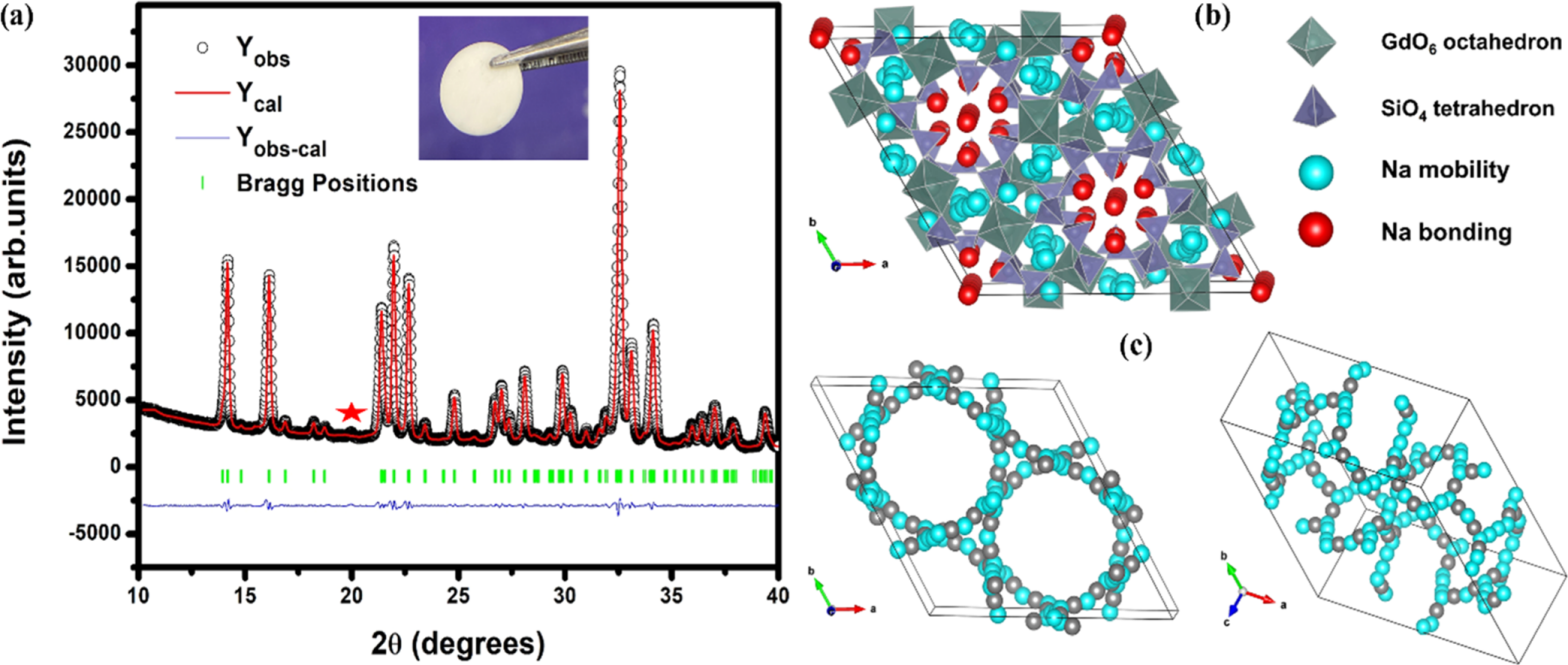
(a) Rietveld refinement of the NGS XRD pattern (* shows the impurity) (inset shows the image of the typical NGS discs synthesized in the second step); (b) crystal structure view of NGS; (c) Na^+^ diffusion paths. Rietveld refinement was done by using FullProf software. The refinement fits and calculated parameters are listed in Table S1. Dots (black color) represent observed data, solid line (red color) represents a calculated pattern, and lower line (blue color) signifies the difference between them. Green-colored vertical lines represent Bragg positions.

In the first attempt, the synthesis of NGS was performed by milling the reactants (strictly stoichiometric and adequately dried) at 400 rpm for 12 h. The milled mixture of reactants was heat-treated at 750 °C for 2 h and then at 1050 °C for 8 h, followed by cooling to RT. The X-ray diffraction (XRD) of the as-synthesized sample, shown in Figure S1, exhibits impurity peaks. To eliminate the impurities, the sample was milled further at 400 rpm for 2 h, pressed into 12 mm discs, heated directly at 1050 °C for 8 h, and cooled to RT. The XRD patterns still revealed the presence of significant impurities. Therefore, in the next step, the milling speed was increased to 600 rpm to reduce the particle size of the reactants further and then repeated the two-step synthesis process. Though such an attempt reduced the intensity of impurities, the presence of impurities was still not eliminated (Figure S2). Previous reports describing the synthesis of compounds belonging to the Na_5_MSi_4_O_12_ family have shown that several other phases exist in the Na–M–Si–O system.^26−28^ Na_3_MSi_3_O_9_ (N3) and Na_9_MSi_6_O_18_ (N9) were reported to be found often during the synthesis of N5-type materials. Generally, N3 is observed at lower temperatures (∼700 °C), and N9 starts to form at ∼950 °C. N3 and N9 types change to N5-type (NGS) structures at higher temperatures. Therefore, we quenched the sample from 1050 °C to stabilize the HT N5 phase. This time, we achieved an almost pure phase of NGS. A minor impurity (shown with an asterisk in Figure 1a) was observed, however, implying the presence of an insignificant amount of N9-type phase. This impurity phase could entirely be avoided through the effective quenching of the sample, for instance, by quenching directly into liquid nitrogen or water. However, due to safety reasons, we have evaded this attempt. NGS samples were prepared on a 5 g scale, and the synthesis was reproduced several times. It should be noted that raising the temperature above 1050 °C resulted in the deformation or melting of the pellets. Other compounds in the Na_5_MSi_4_O_12_ series could also be prepared following this method and will be reported in the future.

Figure 1a shows the Rietveld refined XRD pattern of NGS obtained by quenching. The refined parameters are given in Table S1. It can be elucidated that the NGS crystallizes in a hexagonal form with a space group of *R*3̅*c* (no. 167). The lattice parameters are *a* = *b* = 21.9865 and *c* = 12.5992 Å, and the volume is *V* = 5274.6 Å^3^ (*Z* = 6). The crystal structure view of the NGS is shown in Figure 1b. It can be inferred that the crystal structure is built up of SiO_4_ tetrahedra, GdO_6_ octahedra, NaO_4_ tetrahedra, and NaO_6_ octahedra. SiO_4_ tetrahedra form Si_12_O_36_ rings running parallel to the *c*-axis, leaving the channels. The GdO_6_, NaO_4_, and NaO_6_ moieties occupy these channels. The red Na atoms are rigid and tightly bonded, while the pale blue Na atoms are highly mobile.

**Figure 2:**
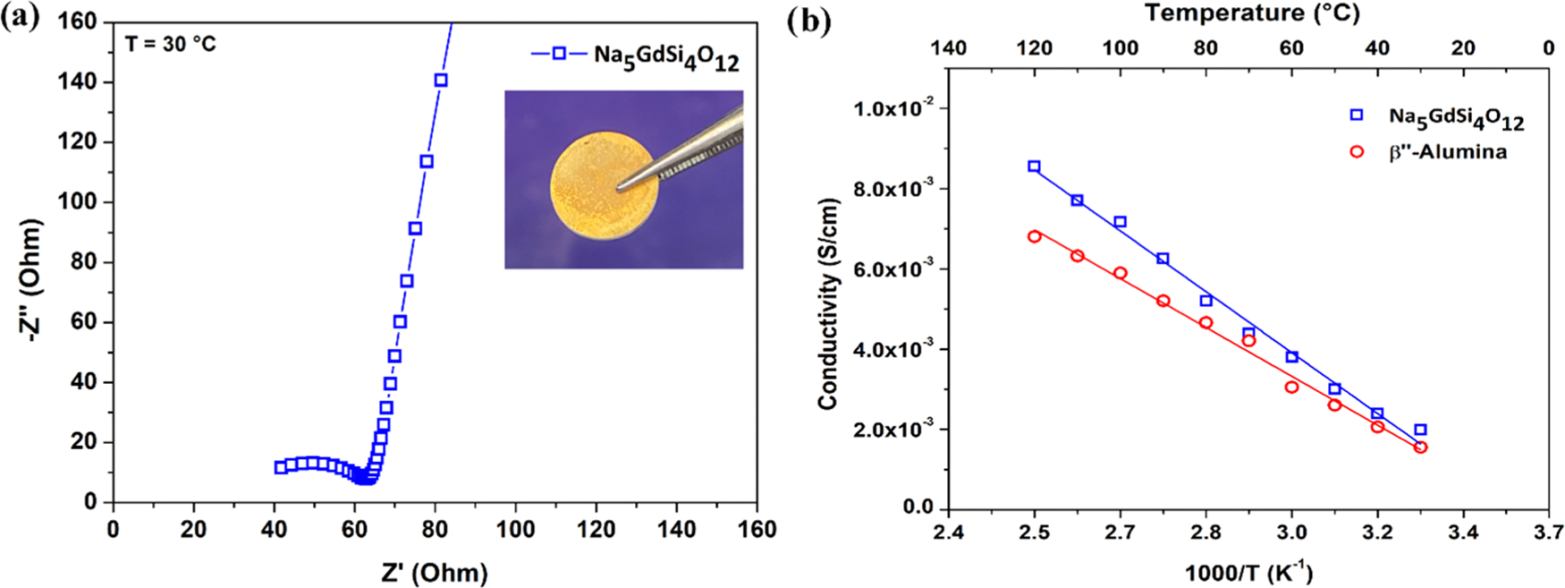
(a) Impedance spectra of NGS obtained at 30 °C (inset shows the typical gold-coated NGS used for ionic conductivity measurements); (b) Arrhenius plot for the temperature-dependent ionic conductivity of NGS and BASE. BASE discs of 12 mm diameter and 1 mm thick and NGS discs of 11 mm diameter and 1.31 mm thick were used for EIS studies. The total resistance, *R*, was determined by calculating the intercept value of the small semicircle with the *X*-axis. The ionic conductivity (σ) was then determined by using σ = *d*/(*R* × *A*), where *R* is resistance, *d* is the thickness, and *A* is the area of the pellet.

Figure 2a shows a Nyquist plot of NGS at 30 °C. The ionic conductivity of NGS at 30 °C is 1.9 × 10^–3^ S cm^–1^ and attained 8.5 × 10^–3^ S cm^–1^ at 120 °C. The ionic conductivity of BASE is 1.5 × 10^–3^ S cm^–1^ and increased to 6.8 × 10^–3^ S cm^–1^ at 120 °C. Thus, the ionic conductivities of NGS are slightly better than BASE. Figure 2b shows the Arrhenius plot of the ionic conductivity of NGS and BASE. The ionic conductivity of NGS could be improved slightly if the impurity in the final sample was eliminated, as we found that impurities due to the N3 and N9 phases can reduce the ionic conductivity of NGS. Figure S3 presents the impedance spectra of pure NGS and the sample synthesized in the first attempt (this sample contains a significant amount of N3 and N9 phases) at 30 °C. The pure sample exhibits higher conductivity.

## Effect of Doping

**Figure 3:**
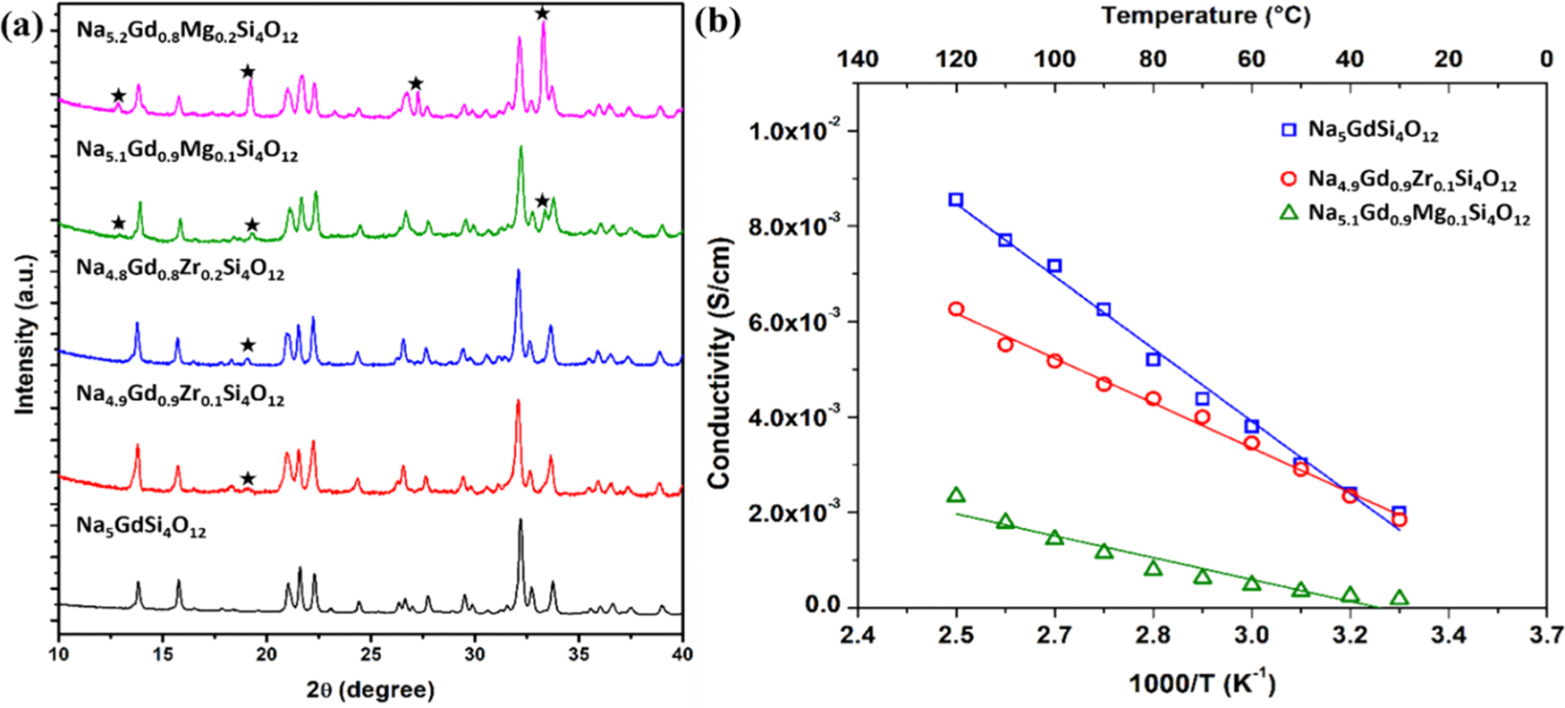
(a) XRD patterns of pure, Zr^4+^, and Mg^2+^ doped NGS; (b) Arrhenius plot for their ionic conductivity of Na_5_GdSi_4_O_12_, Na_4.9_Gd_0.9_Zr_0.1_Si_4_O_12_, and Na_5.1_Gd_0.9_Mg_0.1_Si_4_O_12_.

An attempt has been made to stabilize and improve the ionic conductivity of NGS by partly substituting Gd^3+^ with aliovalent Zr^4+^ and Mg^2+^. The Zr^4+^ and Mg^2+^ ions were chosen as dopants due to their identical ionic radii (0.72 Å) and proven electrochemical stability. It has been observed that Zr^4+^ doping has led to Na-deficient phases (Na_4.9_Gd_0.9_Zr_0.1_Si_4_O_12_ and Na_4.8_Gd_0.8_Zr_0.2_Si_4_O_12_), while Mg^2+^ doping has led to Na-rich phases (Na_5.1_Gd_0.9_Mg_0.1_Si_4_O_12_ and Na_5.2_Gd_0.8_Mg_0.2_Si_4_O_12_). Indeed, it must be emphasized that the Zr^4+^ doping stabilized the NGS phase by exterminating the formation of the N9-type phase. However, a new impurity was found to appear in Na_4.9_Gd_0.9_Zr_0.1_Si_4_O_12_, which grew in the Zr_0.2_ phase (Figure 3a). The Mg^2+^ doping also stabilized the NGS phase by eliminating the formation of the N9-type phase. Surprisingly, the N3 type phase was also observed in Mg-doped samples along with the new phase observed in Zr-doped samples (Figure 3a). The ionic conductivity of Na_4.9_Gd_0.9_Zr_0.1_Si_4_O_12_ and NGS is the same at RT. However, the Zr-doped sample showed a higher resistance as the temperature increased (Figure 3b). The ionic conductivity of Na_5.1_Gd_0.9_Mg_0.1_Si_4_O_12_ is significantly lower compared to that of pure NGS. This could be due to the Na-rich phase created by Mg doping, which reduces the Na vacancy and conductivity.

## Stability in Water

The stability of solid electrolytes is an important factor as it facilitates the simple fabrication of solid-state batteries and enables aqueous processing.^29^ Therefore, it is crucial to examine the stability of NGS in water. In order to do this, the NGS powder was stirred in water overnight at RT, filtered, dried, and heated to 900 °C. Figure S4 shows the XRD pattern of the NGS recorded after heating. The XRD pattern of the water-treated sample is almost unchanged. Nevertheless, high background noise was observed in the XRD pattern, which eventually disappeared after the heat treatment. The background noise is attributed to the surface amorphization of NGS. Further investigations are underway to test the stability of NGS for deeper insights. Overall, NGS is stable in water.

## Chronopotentiometry Studies

**Figure 4:**
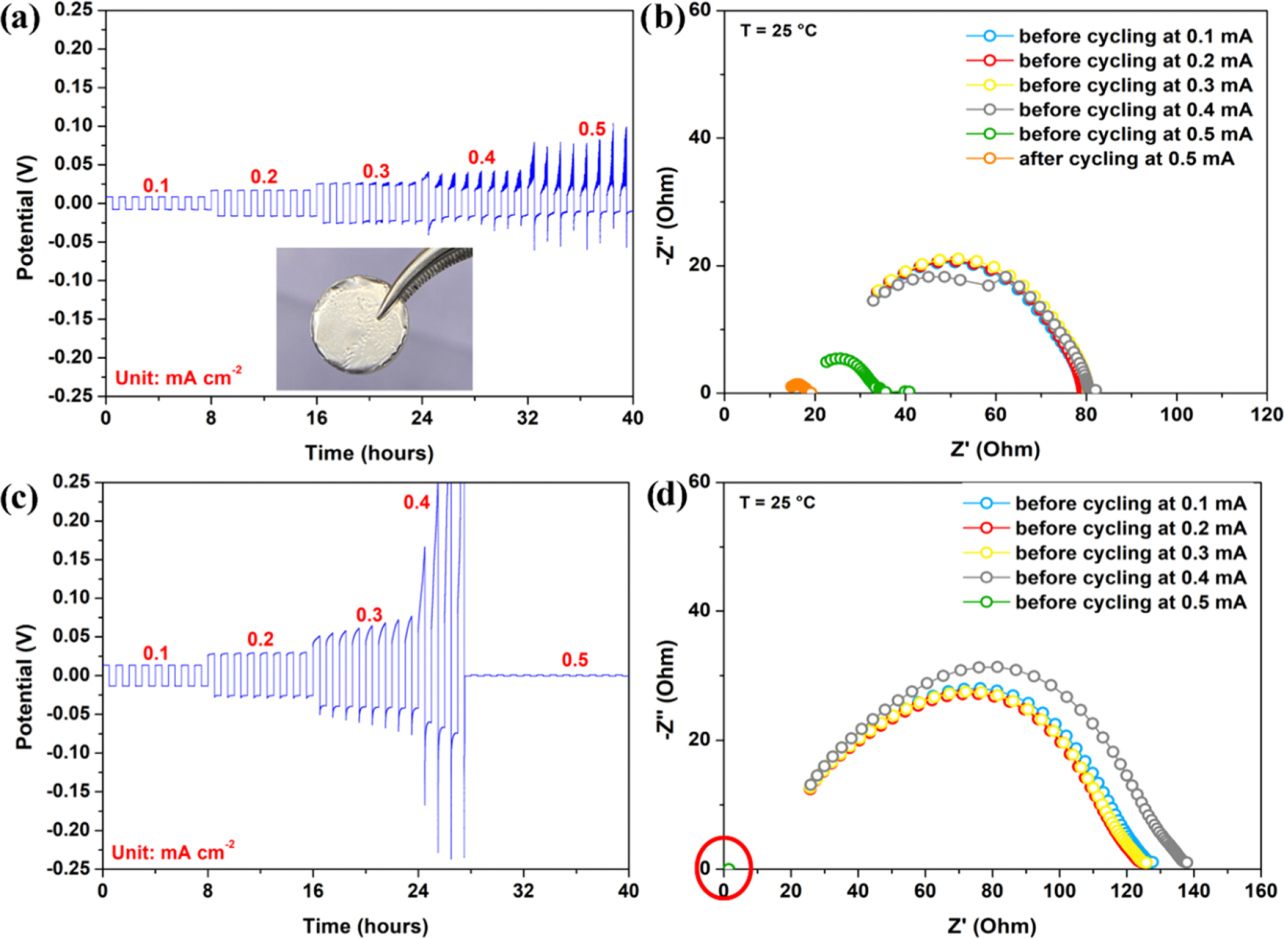
(a, c) chronopotentiometry of NGS and BASE (inset shows the NSG disc sandwiched between aluminum-supported sodium foils); (b, d) EIS spectra of the same cells recorded before the rise of the current in each step.

The ability to deposit and strip Na-metal reversibly at lower potentials and higher current rates is another vital quality of solid electrolytes. We explored this property in NGS and BASE by reversibly plating the Na-metal in symmetrical cells. Figure 4 shows the Na deposition and stripping behavior of NGS and BASE. For measurements, the solid electrolyte discs were sandwiched between aluminum-supported sodium foils (shown in the inset of Figure 4a), transferred to a modified Swagelok cell, and placed between two stainless–steel (SS) current collectors. The top part of the SS current collector was compressed with an SS spring using a Swagelok tightening rod (the modified cell design was given in our previous report^30^). The compression force of the spring was 40 N, measured with a MARK-10 (ES30) force stand. Figure 4a shows the evolution of cell voltage profiles as a function of current measured at 25 °C. The deposition and stripping were carried out for 8 h at each step, and the current was reversed every 0.5 h. The current density was increased stepwise from 0.1 to 0.5 mA cm^–2^ after every 8 h. During the Na deposition and stripping, the Na^+^ ions shuttle through the solid electrolyte and are deposited or stripped from the metal disc. Na-metal is expected to deposit along the grain boundaries of the solid electrolytes during this shuttling (deposition/stripping) process, particularly at higher current rates. This deposition and stripping will be less efficient at higher current rates, leading to dendrite growth and eventually creating short circuits in the cell. Therefore, we measured the interfacial resistance before raising the current in each step to monitor the change in the interfacial resistance and estimate the short-circuit current density (Figure 4a,b). The interfacial resistance indicates the contact resistance between the solid electrolyte and the metal. The lower interfacial resistance reflects better contact between solid electrolytes and Na-metal. In the case of NGS, the interfacial resistance of 80 Ω indicates clean interfaces and the absence of any reaction between NGS and Na-metal. The deposition and stripping potential depends on the contact resistance between the solid electrolytes and metal beyond the ionic conductivity of the solid electrolyte.

Between 0.1 and 0.3 mA cm^–2^, Na/NGS/Na exhibits smoother and more stable deposition. Nevertheless, when the current was raised, the deposition potential increased. A further increase to 0.4 mA cm^–2^ induced the noise in the voltage profile. Surprisingly, the interfacial resistance was reduced to 40 Ω. Further rise in the current increased the noise level in the voltage profile and reduced the interfacial resistance to 20 Ω. A similar trend was observed in several Na/NGS/Na symmetric cells. The deposition, stripping, and resistance behavior of four more Na/NGS/Na cells are shown in Figures S5 and S6. Though the resistance came down at higher current rates, the cells never short-circuited in the current range of 0.1–0.5 mA cm^–2^. We hypothesize that the decrease in the resistance at higher current rates could be due to the Na-metal deposits or dendrites inside the solid electrolytes that lie close. The uneven deposition of Na-metal on the grain boundary surfaces could induce noise in the voltage profiles. However, at this stage, the deposits or dendrites cannot connect electronically and short-circuit, as the pores in the solid electrolytes are disordered (or indirect). Nevertheless, these dendrites would gain more mechanical strength at higher current rates, puncture the pore walls, and lead to short circuits. This is purely a hypothesis postulated based on experimental observation. We are currently trying to understand the pore structure and performing detailed experiments to understand the deposition and stripping behavior in NGS and related compounds.

The deposition and stripping in Na/BASE/Na cells were smoother at all current rates (Figure 4c). The interfacial resistance and the deposition potentials are also higher in Na/BASE/Na cells compared to Na/NGS/Na cells at respective currents. In Na/BASE/Na cells, the potential remained flat at around 13 mV when cycling at 0.1 mA cm^–2^ and increased gradually with current up to 0.3 mA cm^–2^. However, upon increasing the current density to 0.4 mA cm^–2^, the deposition/stripping potential was raised sharply to 0.47 V, followed by a sudden drop to 0 V within a few deposition cycles, indicating short-circuiting of the cell. The resistance dropped to 0 Ω, confirming short-circuiting (Figure 4d). The deposition, stripping, and resistance behavior of an additional Na/BASE/Na cell are shown in Figure S7. This cell also short-circuited between 0.4 and 0.5 mA cm^–2^. We additionally confirmed the short-circuiting with a multimeter continuity test.

**Figure 5:**
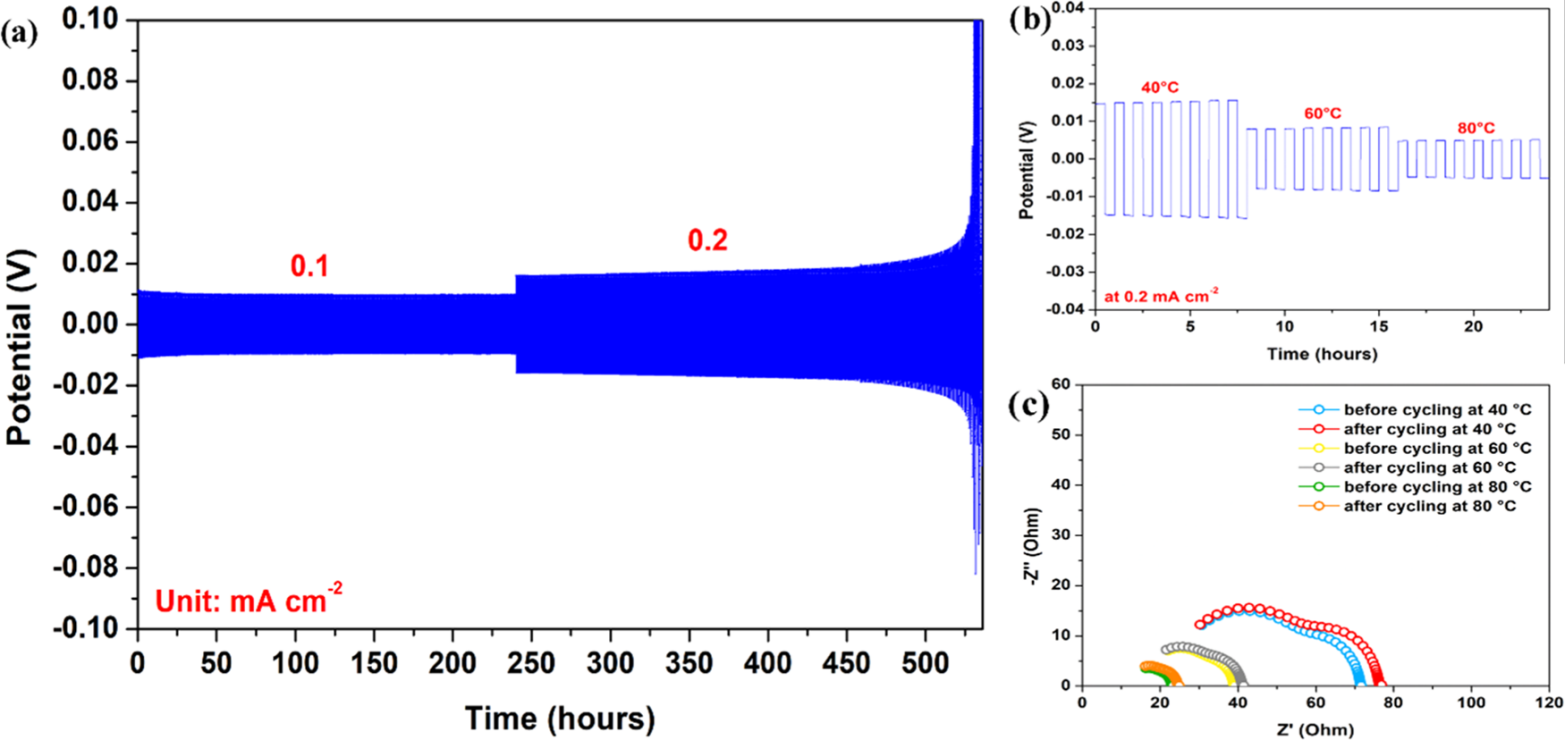
(a) Extended chronopotentiometry studies of Na/NGS/Na cell; (b) chronopotentiometry of Na/NGS/Na cell at various temperatures; (c) EIS spectra of the cell.

Na/NGS/Na cells showed a stable cycling profile with no significant overpotential for almost 23 days (Figure 5a). It also showed a stable cycling profile at elevated temperatures (Figure 5b). The deposition potential decreased with an increase in the temperature, probably due to the improved ionic conductivity of the solid electrolyte at higher temperatures and due to the improved contact between Na and NGS, caused by the improved malleability of Na-metal. Further, this ultralow interfacial resistance of 20 Ω highlights the clean interfaces between NGS and Na-metal, and it has to be noted that there is no reaction even at 80 °C. The chronopotentiometry studies of Na/Na_4.9_Gd_0.9_Zr_0.1_Si_4_O_12_/Na cells are shown in Figure S8. Though the cells showed less overpotential and low initial interfacial resistance, these cells short-circuited between 0.2 and 0.3 mA cm^–2^.

## Solid-State Batteries with NGS

Figure S9(a) shows the charge–discharge curves of Na_0.7_Mn_0.9_Mg_0.1_O_2_ (80 wt %) + Super P (10 wt %) + Na_2_SiO_3_ (10 wt %)/NGS/Na cell at 80 °C. The Na_0.7_Mn_0.9_Mg_0.1_O_2_ was used as a cathode, Super P was used as a conductive additive, and Na_2_SiO_3_ was used as a binder (one of the newly introduced ionically conducting inorganic binders^28^). Na_0.7_Mn_0.9_Mg_0.1_O_2_, Super P, and Na_2_SiO_3_ powders were mixed with an agate mortar and pestle. A thick slurry was made by adding water to the dry mixture. This slurry was coated on NGS solid electrolyte discs dried at RT and heated at 300 °C for 4 h. The discs were then transferred to the glovebox, and the Na disc supported on an aluminum foil was stuck on the other side of the electrolyte, acting as an anode. As prepared, the cells were transferred to a modified Swagelok cell and cycled. The reversible capacity was only 6 mAh g^–1^, which was raised gradually to 12 mAh g^–1^, but then reduced to 8 mAh g^–1^ after 100 cycles. It should be noted that no liquid electrolyte was added to the cathode layer to maintain the safety attributes of all-solid-state batteries. While NGS can be cycled in solid-state batteries, the poor performance was attributed to the low ionic conductivity of the binder. The symmetric Na/NGS/Na cell showed very low impedance of only 80 Ω (Figure 4b), whereas the EIS spectra of the Na_0.7_Mn_0.9_Mg_0.1_O_2_ (80 wt %) + Super P (10 wt %) + Na_2_SiO_3_ (10 wt %)/NGS/Na cell showed extremely high impedance of over 90000 Ω Figure S9(b). This could be due to the poor ionic conductivity of the binder.^31^ We expect to improve the performance of SSSBs by using high-conducting binders.

## Conclusions

In conclusion, we established a simple two-step process to synthesize highly pure NGS. Following this method, we could synthesize several other highly conducting compounds in this series. Further optimization of the synthesis process is possible. We also showed that aliovalent cation doping could be used to stabilize the high-temperature NGS phase. However, doping reduced the ionic conductivity. NGS showed high ionic conductivity, chemical as well as electrochemical stability, and stability in water. More importantly, NGS showed ultralow interfacial resistance of 80 Ω at 25 °C and reduced to 20 Ω at 80 °C, demonstrating highly clean and stable interfaces between NGS and Na-metal. Unequivocally, NGS outperformed BASE in terms of key solid electrolyte properties. Though the synthesis of NGS is simple and can be synthesized at a lower temperature than BASE, the final cost of NGS might be higher than that of BASE due to the presence of Gd. Gd is relatively expensive and low abundant in the earth’s upper crust (4 ppm).^32^ Currently, we are investigating more sustainable compounds in the Na_5_MSi_4_O_12_ series, which could potentially replace BASE in all aspects.

## Experimental Section

### Synthesis and Characterization

Starting materials Na_2_CO_3_ (99.5%, Alfa Aesar), Gd_2_O_3_ (99.9%, Thermo Scientific Chemicals), and SiO_2_ (99.5%, Thermo Scientific Chemicals) were dried before use. For doped NGS compounds, ZrO_2_ (99.7%, Thermo Scientific Chemicals) and MgO (99.95%, Thermo Scientific Chemicals), also dried before use, were added to the starting mixture. Fritsch Pulverisette 6 was used for ball milling experiments (ZrO_2_ vials (80 mL), and balls were used for milling). The ball-to-powder weight ratio was 10:1. BASE discs of 12 mm diameter and 1 mm thick were obtained from Ionotec Ltd., England. Polycrystalline P2-Na_0.7_Mn_0.9_Mg_0.1_O_2_ (NMO) was synthesized according to the previous report.^33^ The crystal structure and phase purity of synthesized materials were determined via XRD using a Bruker D8 Discover diffractometer and Cu Kα radiation (40 kV; 40 mA). Scans were recorded between 10 and 60°. Rietveld Refinement was performed by using FullProf software.

### Electrochemical Characterization

Electrochemical impedance spectroscopy (EIS) and chronopotentiometry measurements were performed using Gamry 1010E potentiostat. Gold (Au) was sputtered as a blocking electrode on either side of the solid electrolyte discs by using an Agar sputter coater for EIS measurements. EIS was recorded between 2 and 10 MHz at RT-120 °C. Ionic conductivities were obtained by fitting the resulting impedance spectra. For chronopotentiometry measurements, Na-metal discs were freshly prepared for each assembled cell in an Ar-filled glovebox. A clean piece of Na was cut from a rod (Na sticks, covered in a film of protective hydrocarbon oil, 99% Alfa Aesar), then pressed flat, and cut into circular electrodes. Their surface was mechanically cleaned by using a scalpel blade to expose fresh sodium. The Na-metal electrodes were then placed on both sides of the NGS pellet. The other side of Na-discs was covered with Al-foil. The Na/NGS/Na stack was pressed by hand. The modified Swagelok cells assembled inside an MBraun glovebox (O_2_ < 0.5 ppm, H_2_O < 0.5 ppm).

## Supporting Information Available

XRD patterns of the samples synthesized via different routes; XRD patterns of water-stirred compounds; chronoamperometry studies on the excess of Na/NGS/Na and Na/BASE/Na cells (PDF)

## References

[ref1] Li F.; Wei Z.; Manthiram A.; Feng Y.; Ma J.; Mai L. Sodium-Based Batteries: From Critical Materials to Battery Systems. J. Mater. Chem. A 2019, 7 (16), 9406–9431. 10.1039/C8TA11999F.

[ref2] Usiskin R.; Lu Y.; Popovic J.; Law M.; Balaya P.; Hu Y.-S.; Maier J. Fundamentals, Status and Promise of Sodium-Based Batteries. Nat. Rev. Mater. 2021, 6 (11), 1020–1035. 10.1038/s41578-021-00324-w.

[ref3] Hwang J.-Y.; Myung S.-T.; Sun Y.-K. Sodium-Ion Batteries: Present and Future. Chem. Soc. Rev. 2017, 46 (12), 3529–3614. 10.1039/C6CS00776G. 28349134

[ref4] Zhao C.; Liu L.; Qi X.; Lu Y.; Wu F.; Zhao J.; Yu Y.; Hu Y.-S.; Chen L. Solid-State Sodium Batteries. Adv. Energy Mater. 2018, 8 (17), 1703012 10.1002/aenm.201703012.

[ref5] Yang H.-L.; Zhang B.-W.; Konstantinov K.; Wang Y.-X.; Liu H.-K.; Dou S.-X. Progress and Challenges for All-Solid-State Sodium Batteries. Adv. Energy Sustainable Res. 2021, 2 (2), 2000057 10.1002/aesr.202000057.

[ref6] Dong Y.; Wen P.; Shi H.; Yu Y.; Wu Z. Solid-State Electrolytes for Sodium Metal Batteries: Recent Status and Future Opportunities. Adv. Funct. Mater. 2023, 2213584 10.1002/adfm.202213584.

[ref7] Li Z.; Liu P.; Zhu K.; Zhang Z.; Si Y.; Wang Y.; Jiao L. Solid-State Electrolytes for Sodium Metal Batteries. Energy Fuels 2021, 35 (11), 9063–9079. 10.1021/acs.energyfuels.1c00347.

[ref8] Fertig M. P.; Skadell K.; Schulz M.; Dirksen C.; Adelhelm P.; Stelter M. From High- to Low-Temperature: The Revival of Sodium-Beta Alumina for Sodium Solid-State Batteries. Batteries Supercaps 2022, 5 (1), e202100131 10.1002/batt.202100131.

[ref9] Will F. G. Effect of Water on Beta Alumina Conductivity. J. Electrochem. Soc. 1976, 123 (6), 834–836. 10.1149/1.2132943.

[ref10] Fertig M. P.; Dirksen C.; Schulz M.; Stelter M. Humidity-Induced Degradation of Lithium-Stabilized Sodium-Beta Alumina Solid Electrolytes. Batteries 2022, 8 (9), 103 10.3390/batteries8090103.

[ref11] Zhang Z.; Wenzel S.; Zhu Y.; Sann J.; Shen L.; Yang J.; Yao X.; Hu Y.-S.; Wolverton C.; Li H.; Chen L.; Janek J. Na_3_Zr_2_Si_2_PO_12_: A Stable Na^+^-Ion Solid Electrolyte for Solid-State Batteries. ACS Appl. Energy Mater. 2020, 3 (8), 7427–7437. 10.1021/acsaem.0c00820.

[ref12] Guin M.; Indris S.; Kaus M.; Ehrenberg H.; Tietz F.; Guillon O. Stability of NASICON Materials against Water and CO_2_ Uptake. Solid State Ionics 2017, 302, 102–106. 10.1016/j.ssi.2016.11.006.

[ref13] Maksimov B. A.; Kharitonov Y. A.; Belov N. V. Crystal structure of the Na-Y metasilicate Na5YSi4O12; Soviet Physics Doklady, 1974; Vol. 18, p 763.

[ref14] Shannon R. D.; Taylor B. E.; Gier T. E.; Chen H. Y.; Berzins T. Ionic Conductivity in Sodium Yttrium Silicon Oxide (Na_5_YSi_4_O_12_)-Type Silicates. Inorg. Chem. 1978, 17 (4), 958–964. 10.1021/ic50182a033.

[ref15] Sivakumaran A.; Samson A. J.; Afroj Bristi A.; Surendran V.; Butler S.; Reid S.; Thangadurai V. High ionic conducting rare-earth silicate electrolytes for sodium metal batteries. J. Mater. Chem. A 2023, 11, 15792–15801. 10.1039/D3TA02128A.

[ref16] Yang A.; Ye R.; Song H.; Lu Q.; Wang X.; Dashjav E.; Yao K.; Grüner D.; Ma Q.; Tietz F.; Guillon O. Pressureless all-solid-state Na/S batteries with self-supporting Na_5_YSi_4_O_12_ scaffolds. Carbon Energy 2023, 5, e371 10.1002/cey2.371.

[ref17] Sun G.; Lou C.; Yi B.; Jia W.; Wei Z.; Yao S.; Lu Z.; Chen G.; Shen Z.; Tang M.; Du F. Electrochemically induced crystalline-to-amorphization transformation in sodium samarium silicate solid electrolyte for long-lasting sodium metal batteries. 2023 10.1038/s41467-023-42308-0. PMC10579357 37845205

[ref18] Hong H. Y. P.; Kafalas J. A.; Bayard M. High Na^+^-Ion Conductivity in Na_5_YSi_4_O_12_. Mater. Res. Bull. 1978, 13 (8), 757–761. 10.1016/0025-5408(78)90037-5.

[ref19] Ahmadzadeh M.; Olds T. A.; Scrimshire A.; Bingham P. A.; McCloy J. S. Structure and Properties of Na_5_FeSi_4_O_12_ Crystallized from 5Na_2_O–Fe_2_O_3_–8SiO_2_ Glass. Acta Crystallogr., Sect. C: Struct. Chem. 2018, 74 (12), 1595–1602. 10.1107/S2053229618014353. 30516142

[ref20] Visco S. J.; Kennedy J. H. Investigation of Na_5_GdSi_4_O_12_(NGS) NASICON and Highly Doped NGS NASICON Prepared by Spray-Freeze/Freeze-Dry Methods Using Complex Plane Analysis. Solid State Ionics 1983, 9–10, 885–889. 10.1016/0167-2738(83)90106-6.

[ref21] Hung L. I.; Wang S. L.; Szu S.; Hsieh C. Y.; Kao H. M.; Lii K. H. Hydrothermal Synthesis, Crystal Structure, Solid-State NMR Spectroscopy, and Ionic Conductivity of Na_5_InSi_4_O_12_, a Silicate Containing a Single 12-Membered Ring. Chem. Mater. 2004, 16 (9), 1660–1666. 10.1021/cm030417e.

[ref22] Fakhar-Bourguiba N.; Gharbi N.; Smiri-Dogguy L.; Boilot J. P. Sol-Gel Preparation, Phase Transition and Ionic Conductivity in Na_5_YSi_4_O_12_ and Na_5_GdSi_4_O_12_. Mater. Res. Bull. 1988, 23 (8), 1185–1191. 10.1016/0025-5408(88)90210-3.

[ref23] Shannon R. D.; Chen H.-Y.; Berzins T. Ionic Conductivity in Na_5_GdSi_4_O_12_. Mater. Res. Bull. 1977, 12 (10), 969–973. 10.1016/0025-5408(77)90020-4.

[ref24] Bentzen J. J.; Nicholson P. S. The Preparation and Characterization of Dense, Highly Conductive Na_5_GdSi_4_O_12_ Nasicon (NGS). Mater. Res. Bull. 1980, 15 (12), 1737–1745. 10.1016/0025-5408(80)90191-9.

[ref25] Wenzel S.; Leichtweiß T.; Weber D.; Sann J.; Zeier W. G.; Janek J. Interfacial Reactivity Benchmarking of the Sodium Ion Conductors Na_3_PS_4_ and Sodium β-Alumina for Protected Sodium Metal Anodes and Sodium All-Solid-State Batteries. ACS Appl. Mater. Interfaces 2016, 8 (41), 28216–28224. 10.1021/acsami.6b10119. 27677413

[ref26] Banks E.; Kim C. H. Ionic Conductivity in Glass and Glass-Ceramics of the Na_3_YSi_3_O_9_ and Na_5_YSi_4_O_12_ Type Materials. J. Electrochem. Soc. 1985, 132 (11), 2617–2621. 10.1149/1.2113635.

[ref27] Yamashita K.; Okura S.; Umegaki T.; Kanazawa T. Synthesis and Ionic Conduction of C3A-Type Nasicon Na_3+3x–y_Y_1–x_Si_3–y_P_y_O_9_. Solid State Ionics 1988, 26, 279–286. 10.1016/0167-2738(88)90255-X.

[ref28] Chen R.; Li Q.; Yu X.; Chen L.; Li H. Approaching Practically Accessible Solid-State Batteries: Stability Issues Related to Solid Electrolytes and Interfaces. Chem. Rev. 2020, 120 (14), 6820–6877. 10.1021/acs.chemrev.9b00268. 31763824

[ref29] Okura T. Development of Na+ Superionic Conducting Na_5_YSi_4_O_12_-Type Glass-Ceramics. Adv. Mater. Lett. 2019, 10 (2), 85–90. 10.5185/amlett.2019.1684.

[ref30] Mohammad I.; Witter R.; Fichtner M.; Reddy M. A. Room-Temperature, Rechargeable Solid-State Fluoride-Ion Batteries. ACS Appl. Energy Mater. 2018, 1, 4766–4775. 10.1021/acsaem.8b00864.

[ref31] Trivedi S.; Pamidi V.; Fichtner M.; Anji Reddy M. Ionically Conducting Inorganic Binders: A Paradigm Shift in Electrochemical Energy Storage. Green Chem. 2022, 24 (14), 5620–5631. 10.1039/D2GC01389D.

[ref32] Dushyantha N.; Batapola N.; Ilankoon I. M.; Rohitha S.; Premasiri R.; Abeysinghe B.; Ratnayake N.; Dissanayake K. The Story of Rare Earth Elements (REEs): Occurrences, Global Distribution, Genesis, Geology, Mineralogy and Global Production. Ore Geol. Rev. 2020, 122, 103521 10.1016/j.oregeorev.2020.103521.

[ref33] Pamidi V.; Trivedi S.; Behara S.; Fichtner M.; Reddy M. A. Micron Sized Single Crystal Cathodes for Sodium-Ion Batteries. iScience 2022, 25, 104205 10.1016/j.isci.2022.104205. 35494248 PMC9043968

