# Supplementary material for: Reinvestigation of Na5GdSi4O12: A Potentially Better Solid Electrolyte than Sodium β Alumina for Solid-State Sodium Batteries
Source: ACS Appl Mater Interfaces. 2024 Jan 31;16(6):7112–8. doi: 10.1021/acsami.3c16153 (PMC10875635; doi:10.1021/acsami.3c16153)
Supplement: Supplementary file 1 — am3c16153_si_001.pdf [file am3c16153_si_001.pdf]

Supporting Information for

Reinvestigation of $Na_5GdSi_4O_{12}$: A Potentially Better Solid Electrolyte than Sodium Beta Alumina for Solid-State Sodium Batteries

Anna Michalak[a], Santosh Kumar Behara[a], and M. Anji Reddy[a, *]

[a]IMPACT Energy Storage Laboratory, Faculty of Science and Engineering, Swansea University, Fabian Way, Swansea SA1 8EN, UK

*Corresponding author Email: a.r.munnangi@swansea.ac.uk

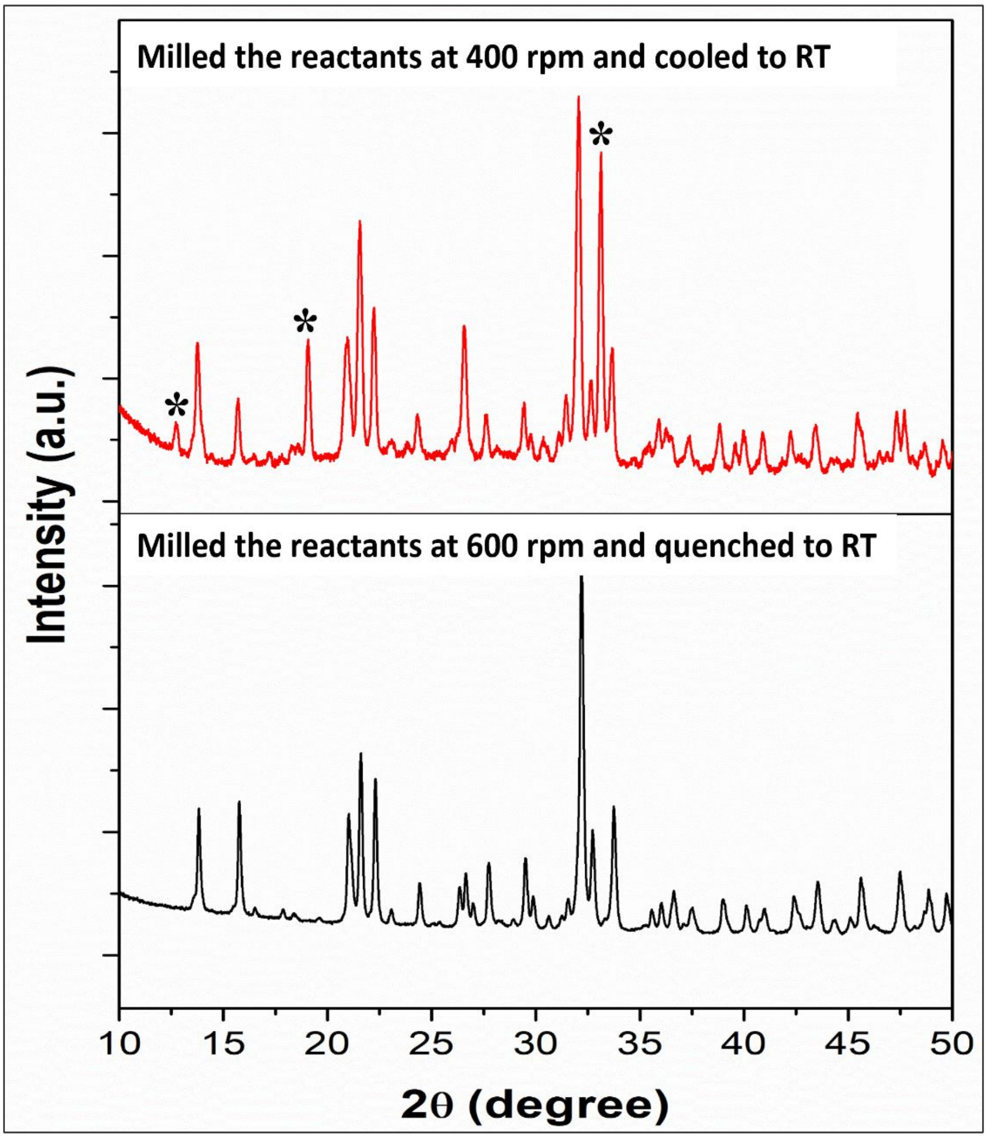

Figure S1 XRD patterns of the samples synthesized via the first and second processes.

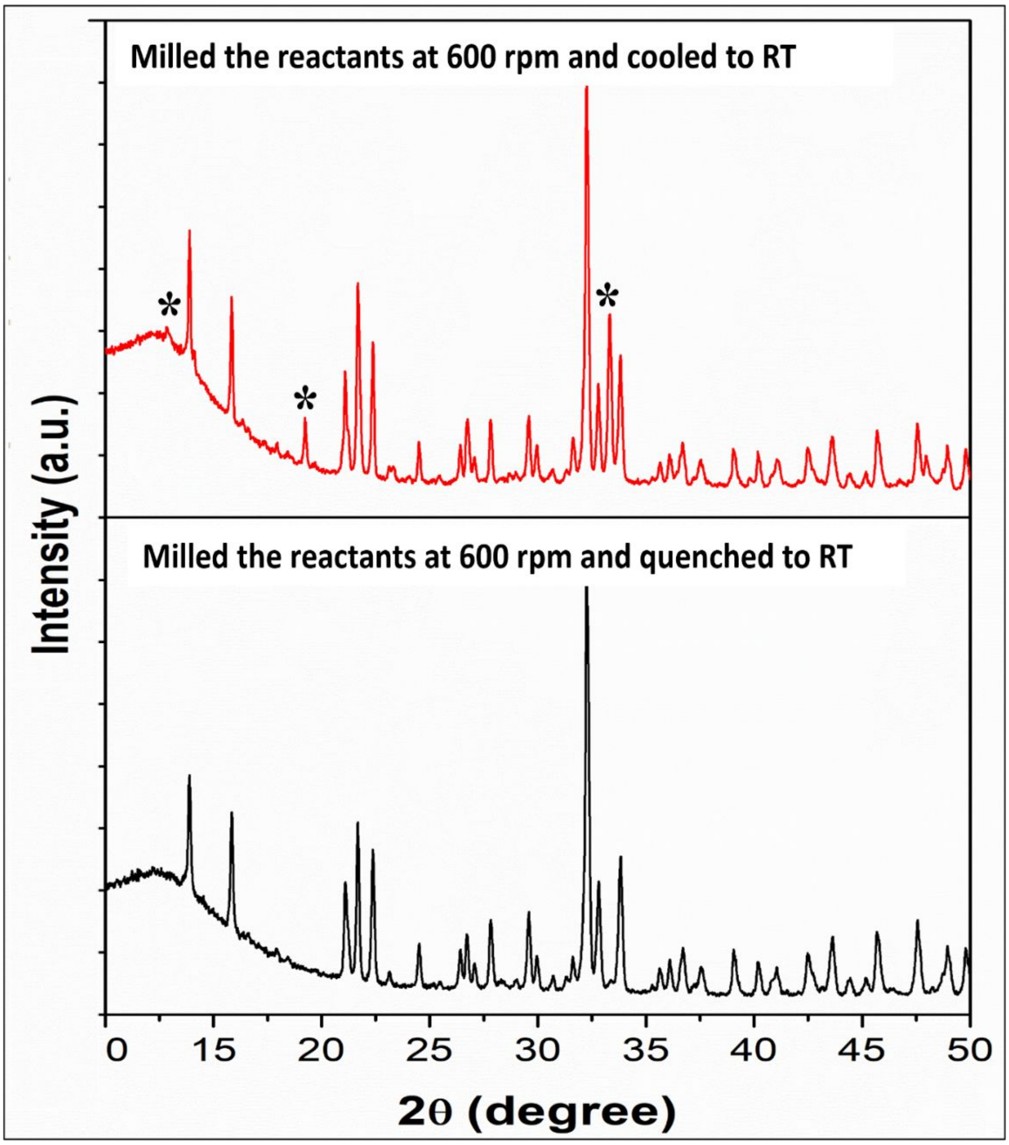

Figure S2 XRD patterns of the samples synthesized via the second process (the difference is one sample cooled to RT and one quenched to RT).

Table S1. Crystallographic data of $Na_5GdSi_4O_{12}$.

**Crystal System**			Hexagonal		
**Space Group**			R-3c (no.167)		
**Lattice Parameter**			a = b = 21.98657 Å, c = 12.59926 Å		
**Volume, Z**			V = 5274.613 Å^3, Z = 6		
**Atoms**	**Wyckoff positions**	**x**	**Y**	**z**	**Occupancy**
Gd1	18*e*	0	0.7503(1)	0.7500(0)	0.4968(1)
Si1	36*f*	0.8953(5)	0.5427(6)	0.3673(5)	1.0000(0)
Si2	36*f*	0.5793(0)	0.1520(8)	0.7106(1)	1.0000(0)
O1	36*f*	0.9038(6)	0.5864(6)	0.2672(4)	1.0000(0)
O2	36*f*	0.0643(9)	0.2003(8)	0.0004(6)	1.0000(0)
O3	36*f*	0.2384(6)	0.6085(3)	0.2900(2)	1.0000(0)
O4	36*f*	0.9110(5)	0.2752(0)	0.2533(1)	1.0000(0)
O5	36*f*	0.8901(3)	0.4755(0)	0.1026(4)	1.0000(0)
O6	36*f*	0.8836(9)	0.3732(2)	0.2186(5)	1.0000(0)
Na1	36*f*	0.0774(7)	0.1494(2)	0.8754(4)	0.8704(5)
Na2	6*b*	0	0	0	0.1949(7)
Na3	6*a*	0	0	0.2500(0)	0.1488(0)
Na4	18*e*	0.2714(8)	0	0.7500(0)	0.4941(5)
Na5	36*f*	0.3313(9)	0.1800(8)	0.3105(4)	0.1354(8)
Na6	36*f*	0.3363(2)	0.1627(7)	0.1068(2)	0.5260(3)

**Anisotropic Betas *10^{-4}**						
**Atoms**	**B11**	**B22**	**B33**	**B12**	**B13**	**B23**
Gd1	33.4	-1.3	-92.8	16.7	25.3	12.6
Si1	-48.4	8.1	37.2	18.2	-22.2	-12.6
Si2	-21.1	-12.6	-17.1	-11.0	29.7	7.8
O1	-110.8	195.3	324.1	10.8	-155.2	-249.6
O2	-3.8	5.5	-7.6	-6.8	57.1	41.4
O3	34.3	231.6	-41.0	76.3	76.5	137.1
O4	-103.8	-67.9	32.2	-120.8	-87.7	53.2
O5	-44.0	-69.4	59.3	-46.9	-26.9	32.9
O6	147.8	91.4	-16.1	101.1	-52.2	123.7
Na1	-102.2	-4.3	141.1	-6.7	114.2	190.3
Na2	-177.9	-177.9	935.6	-89.0	0	0
Na3	-81.3	-81.3	-85.3	-40.6	0	0
Na4	-43.4	81.5	-179.6	40.8	-64.1	-128.1
Na5	284.8	580.8	-143.5	-134.8	-459.5	187.3
Na6	-452.5	644.7	-221.2	-185.5	210.5	172.5

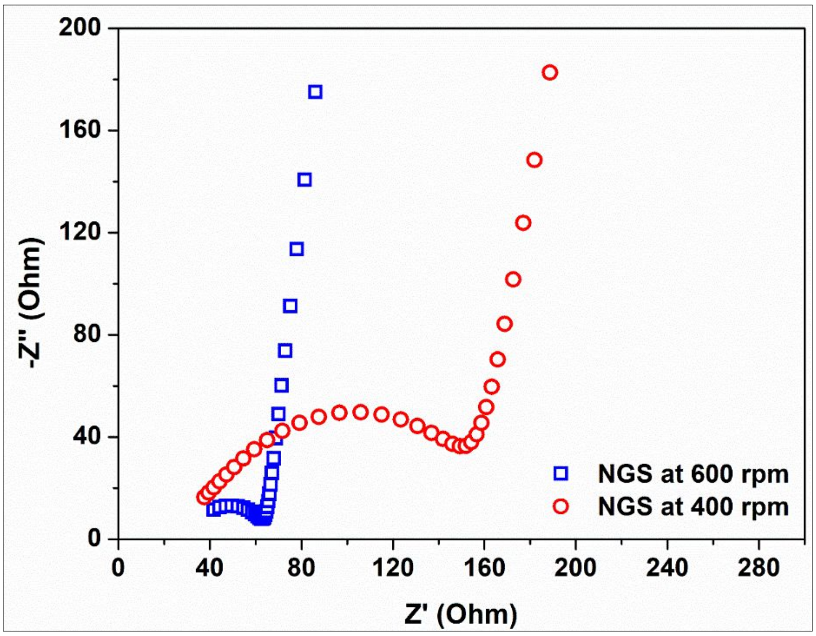

Figure S3 Impedance spectra of the sample synthesized in the first attempt (this sample contains a significant amount of Na3 and Na9 phases) compared with pure NGS at 30 °C.

Table S2 Ionic conductivities of BASE, NGS, $Na_{4.9}Gd_{0.9}$Zr0.1Si_4O_{12},$Na_{5.2}Gd_{0.8}Zr_{0.2}Si_4O_{12}$ and $Na_{5.1}Gd_{0.9}Mg_{0.1}Si_4O_{12}$.

T (°C)	Ionic Conductivity (S cm^{-1})				
	**$Na_5GdSi_4O_{12}$**	**Na-β” Alumina**	**$Na_{4.9}Gd_{0.9}Zr_{0.1}Si_4O_{12}$**	**$Na_{5.2}Gd_{0.8}Zr_{0.2}Si_4O_{12}$**	**$Na_{5.1}Gd_{0.9}Mg_{0.1}Si_4O_{12}$**
30	1.9 x 10^{-3}	1.5 x 10^{-3}	1.8 x 10^{-3}	4.6 x 10^{-4}	1.8 x 10^{-4}
40	2.4 x 10^{-3}	2.0 x 10^{-3}	2.3 x 10^{-3}	6.3 x 10^{-4}	2.5 x 10^{-4}
50	3.0 x 10^{-3}	2.6 x 10^{-3}	2.9 x 10^{-3}	8.5 x 10^{-4}	3.5 x 10^{-4}
60	3.8 x 10^{-3}	3.0 x 10^{-3}	3.5 x 10^{-3}	1.1 x 10^{-3}	4.8 x 10^{-4}
70	4.3 x 10^{-3}	4.2 x 10^{-3}	4.0 x 10^{-3}	1.4 x 10^{-3}	6.3 x 10^{-4}
80	5.2 x 10^{-3}	4.6 x 10^{-3}	4.4 x 10^{-3}	1.8 x 10^{-3}	7.9 x 10^{-4}
90	6.2 x 10^{-3}	5.2 x 10^{-3}	4.7 x 10^{-3}	2.3 x 10^{-3}	1.1 x 10^{-3}
100	7.1 x 10^{-3}	5.9 x 10^{-3}	5.2 x 10^{-3}	2.6 x 10^{-3}	1.4 x 10^{-3}
110	7.7 x 10^{-3}	6.3 x 10^{-3}	5.5 x 10^{-3}	3.2 x 10^{-3}	1.8 x 10^{-3}
120	8.5 x 10^{-3}	6.8 x 10^{-3}	6.3 x 10^{-3}	3.7 x 10^{-3}	2.3 x 10^{-3}

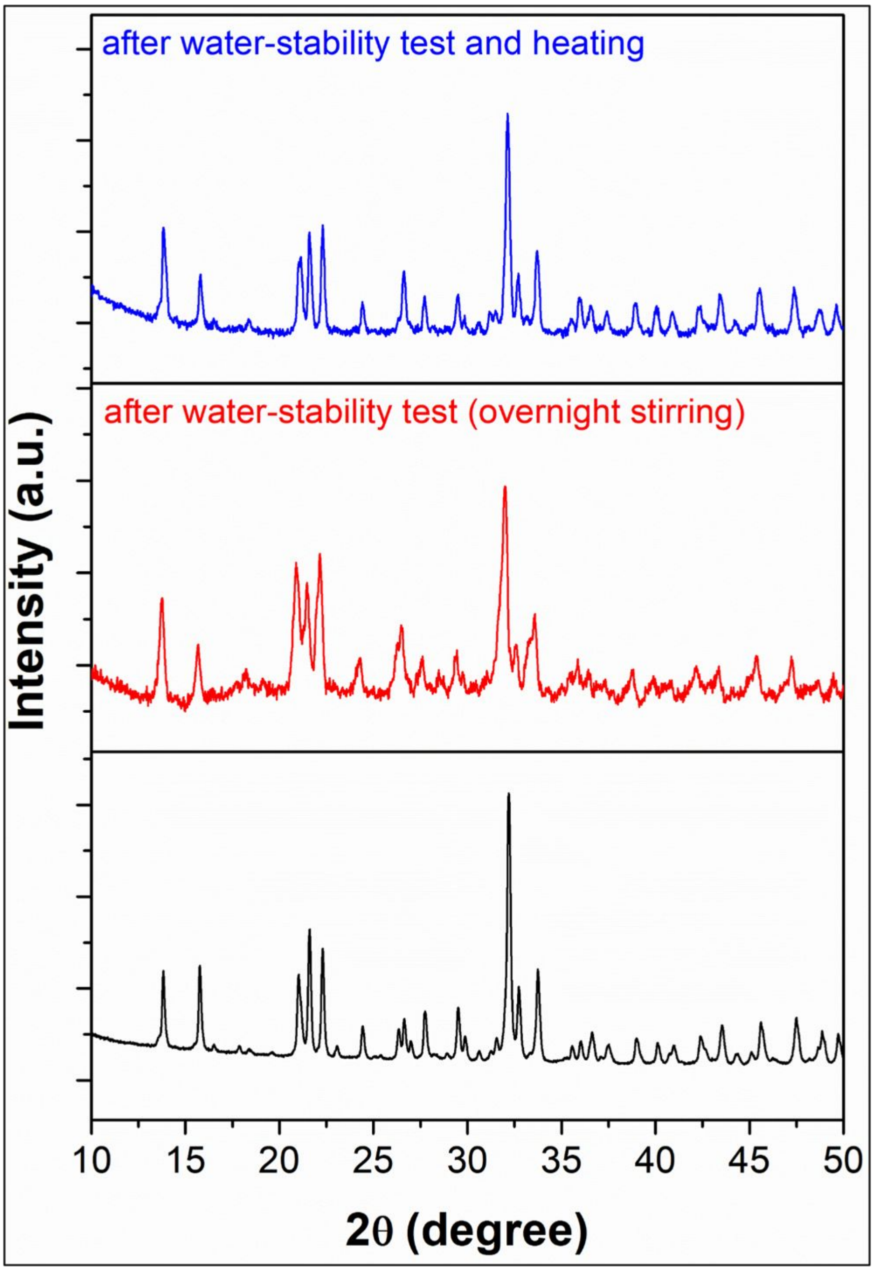

Figure S4 XRD patterns of the samples stirred in water (red) and dried and heated at 900 °C (blue), compared with pure sample.

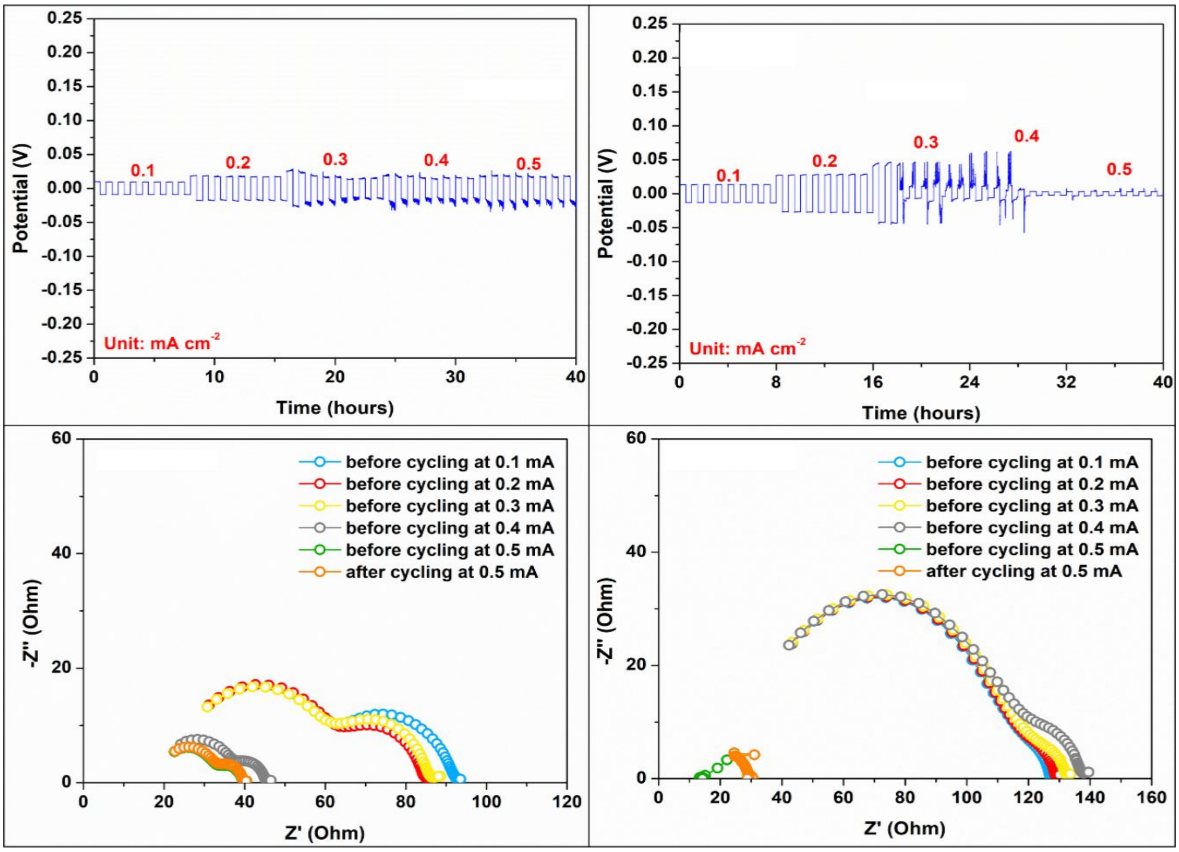

Figure S5 Chronopotentiometry and impedance spectra of 2nd and 3rd Na/NGS/Na cells

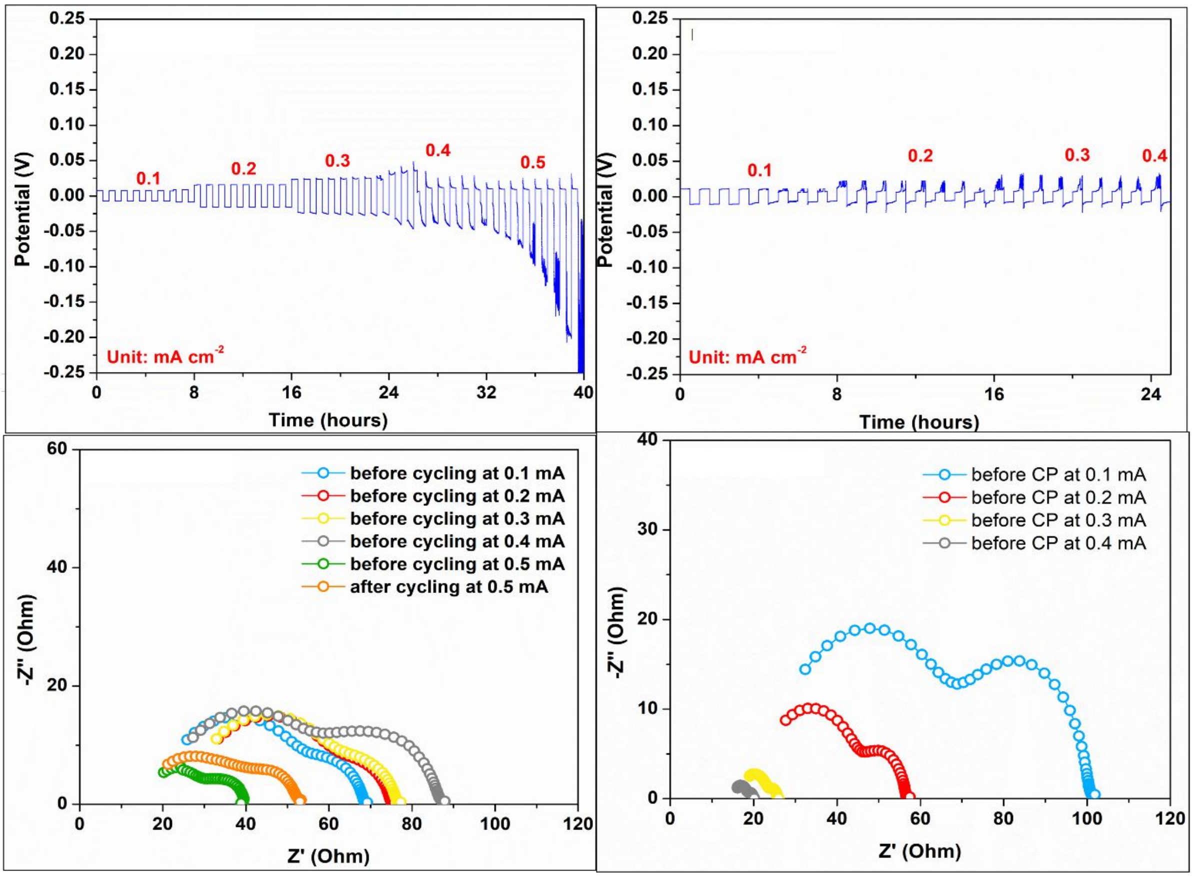

Figure S6 Chronopotentiometry and impedance spectra of 4th and 5th Na/NGS/Na cells.

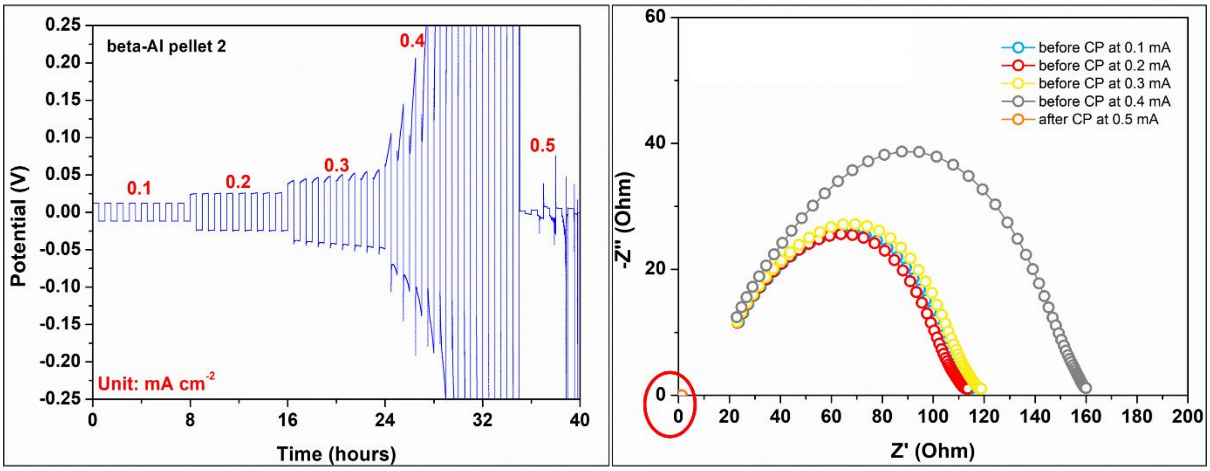

Figure S7 Chronopotentiometry and impedance spectra of Na/SBA/Na cell.

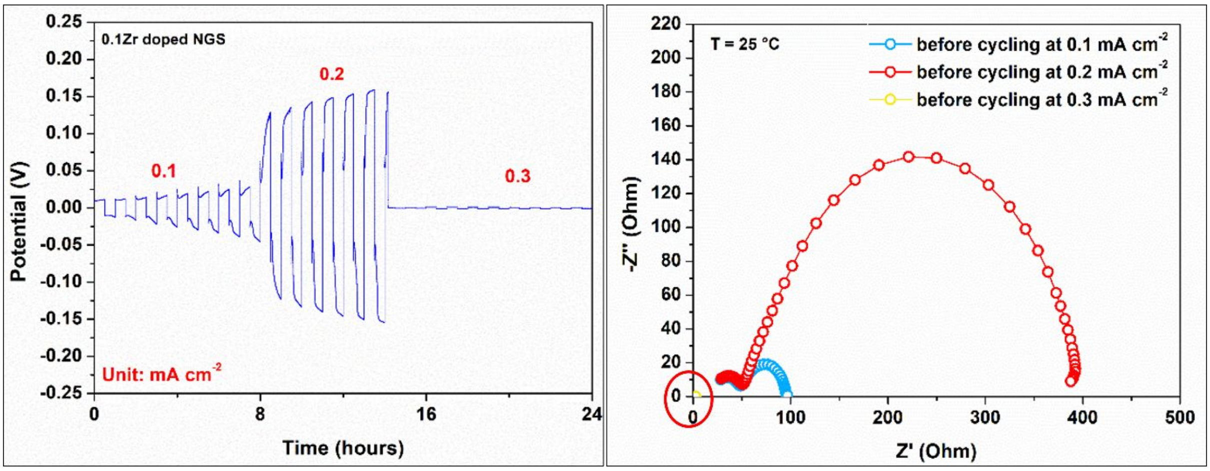

Figure S8 Chronopotentiometry and impedance spectra of Na/ $Na_{4.9}Gd_{0.9}Zr_{0.1}Si_4O_{12}$ /Na cell.

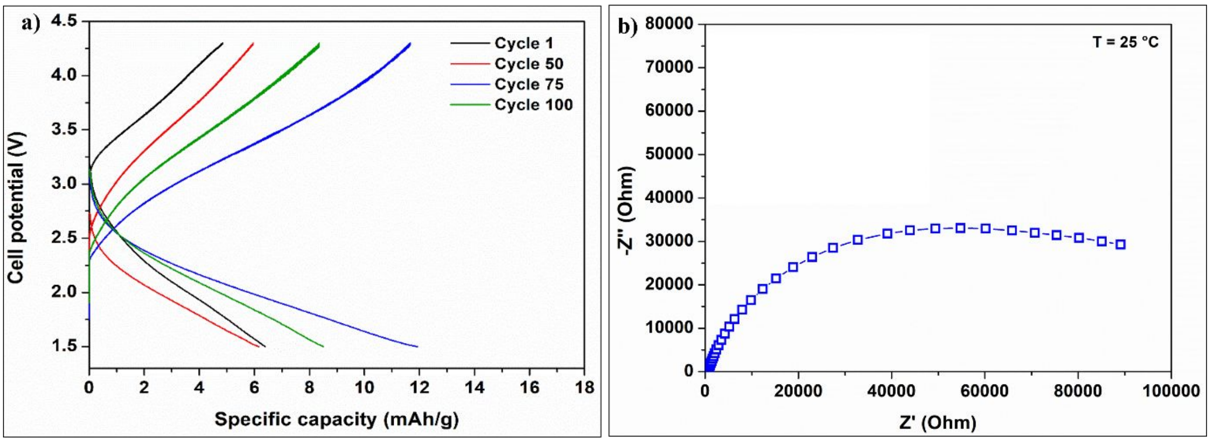

Figure S9 (a) charge discharge curves of $Na_{0.7}Mn_{0.9}Mg_{0.1}O_2$ (80wt%) + Super P (10wt%) + Na_2SiO_3 (10wt%) /$Na_5GdSi_4O_{12}$ /Na cell (without liquid electrolyte) at 80 °C; (b) EIS spectra of the cell.
